# A Lethal Case of* Sphingomonas paucimobilis* Bacteremia in an Immunocompromised Patient

**DOI:** 10.1155/2016/3294639

**Published:** 2016-03-20

**Authors:** Nata Pratama Hardjo Lugito, Cucunawangsih, Andree Kurniawan

**Affiliations:** ^1^Department of Internal Medicine, Faculty of Medicine, Pelita Harapan University, Jendral Sudirman Boulevard, Lippo Karawaci, Tangerang, Banten 15811, Indonesia; ^2^Department of Microbiology, Faculty of Medicine, Pelita Harapan University, Jendral Sudirman Boulevard, Lippo Karawaci, Tangerang, Banten 15811, Indonesia

## Abstract

*Sphingomonas paucimobilis* is a yellow-pigmented, glucose nonfermenting, aerobic, Gram negative bacillus of low pathogenicity. This organism was found in the implantation of indwelling catheters, sterile intravenous fluid, or contaminated hospital environment such as tap and distilled water, nebulizer, ventilator, and hemodialysis device. A 55-year-old female was hospitalized for diabetic foot ulcer in the presence of multiple comorbidities: diabetes mellitus, colonic tuberculosis, end-stage renal disease, and indwelling catheters for central venous catheter and hemodialysis. The patient passed away on the 44th day of admission due to septic shock. The organism found on blood culture on the 29th day of admission was multidrug resistant* S. paucimobilis*. Severe infection and septic shock due to* S. paucimobilis* have been reported particularly in immunocompromised patients, but there has been only one reported case of death in a premature neonate with septic shock. This is the first reported lethal case of* S. paucimobilis* bacteremia in an adult patient.

## 1. Introduction

*Sphingomonas paucimobilis* is a Gram negative bacillus of low pathogenicity [1–3]. Human can acquire it in community or hospital settings [4–6]. Severe infection and septic shock due to* S. paucimobilis* have been reported particularly in immunocompromised patients [2, 4, 7], but only one case of death in a premature neonate has been reported [8]. In hospital setting, this organism was found in implantation of indwelling catheters, sterile intravenous fluid, or contaminated hospital environment such as tap and distilled water, nebulizer, ventilator, and hemodialysis device [6, 9] and isolated from various clinical specimens [10].* S. paucimobilis* is usually susceptible to carbapenems, aminoglycosides, trimethoprim sulfamethoxazole, and piperacillin/tazobactam and resistant to penicillins and first-generation cephalosporins. We report a lethal case of an immunocompromised adult patient with* S. paucimobilis* bacteremia that was resistant to the antibiotics; it is usually susceptible to aminoglycosides, fluoroquinolones, trimethoprim sulfamethoxazole, and some third-generation cephalosporins.

## 2. Case Report

A 55-year-old female came to the emergency unit with an ulcer on the sole of the left foot. One month prior to presentation, it was only a small blister, but after a week the injury began to ulcerate. She experienced fever and decreased appetite one week before. The patient had long-standing history of uncontrolled diabetes since 5 years ago. On physical examination, she was moderately ill, compos mentis, with normal blood pressure, tachycardia, tachypnea, and fever. General examinations were within normal limit. On the sole of the left foot there was an ulcer measuring 6 × 5 centimeters. The ulcer was dirty and odorous. Laboratory examination showed leucocyte count 23,000/*μ*L with 86% neutrophil, random blood glucose, 354 mg/dL, keton, 0.9 mg/dL, urea, 259 mg/dL, creatinine, 5.8 mg/dL, proteinuria (++), and metabolic acidosis in blood gas analysis. Other laboratory examinations, chest radiology, and electrocardiography were within normal range. Her foot radiology showed porous structure, lytic lesion on calcaneus bone, and subcuticular emphysema. The patient was diagnosed with sepsis due to diabetic foot ulcer, diabetic ketoacidosis, and end-stage renal disease. The patient was treated with intravenous insulin drip of 1 unit/hour and intravenous empiric antibiotics of ceftriaxone, 2 grams two times a day, and metronidazole, 500 mg three times a day. Below knee amputation and wound debridement were done on the 2nd day of admission. She was admitted to intensive care unit and undergone hemodialysis 2 times per week.

On the 7th day of admission, she experienced dyspnea and rales on both fields of the lung. There were new left paracardial infiltrates on chest radiology. Blood gas analysis showed respiratory acidosis and hypoxemia. The patient was diagnosed with acute respiratory distress syndrome due to hospital acquired pneumonia and then intubated and put on mechanical ventilation. The previous antibiotic therapies were substituted with intravenous piperacillin tazobactam, 4.5 grams four times a day, amikacin, 1 gram every 48 hours, fluconazole, 150 mg once daily, and peroral trimethoprim sulfamethoxazole, 960 mg two times a day. Chest radiology on the 14th day of admission showed improvement, the blood cultures for bacteria and fungus were negative. But then, on the 19th day, the chest radiology worsened, showing supraclavicular, perihilar, and pericardial infiltrates. Sputum culture on the 22nd day showed* Acinetobacter baumanii* that was susceptible to trimethoprim sulfamethoxazole, amikacin, and fosfomycin, but the blood culture was negative. Patient was treated accordingly with intravenous amikacin, 1 gram every 48 hours, fosfomycin, 500 mg once daily, and peroral trimethoprim sulfamethoxazole, 960 mg two times a day.

On the 14th day, the patient experienced hematochezia, and colonoscopy was done. The colonoscopy showed multiple ulcer on rectum and sigmoid of low density, a segment of ulceroglandular lesion easily bleeding on the proximal descending colon suggestive of colonic tuberculosis. Patient was treated with peroral rifampicin, 450 mg, isoniazid, 300 mg, and pyrazinamide and ethambutol, 1,000 mg per day, and 12 days later the hematochezia resolved.

Blood culture from the 29th day showed* Sphingomonas paucimobilis* that was susceptible to cefoperazone, cefoperazone sulbactam, imipenem, and tigecycline. As the result came out on the 44th day of admission, antibiotic treatment was not changed according to the result. The patient's condition deteriorated since the 35th day. She had septic shock and was put on intravenous norepinephrine and dobutamine to maintain mean arterial pressure above 65 mmHg. On the 44th day of admission, the patient passed away with irreversible septic shock as the cause of death. The result of blood and sputum cultures was shown in Table 1.

## 3. Discussion

*Sphingomonas paucimobilis* is a yellow-pigmented, glucose nonfermenting, aerobic, oxidase-positive, Gram negative bacillus [1, 2]. In the* Sphingomonas* genus,* S. paucimobilis* is regarded as the main pathogenic species [3]. This organism can be found in soil or water, so human can acquire it in community or hospital settings [4–6]. It has been isolated from a wide variety of clinical specimens including blood, urine, sputum, and cerebrospinal fluid [10]. The blood culture of the patient on the 22nd day of admission was sterile and then the blood culture on the 29th day was positive for* S. paucimobilis*, showing that the bacteremia was a hospital infection. In hospital settings, such as in this case report,* S. paucimobilis* could have originated from devices such as implantation of indwelling catheters, sterile intravenous fluid, or contaminated hospital environment such as tap and distilled water, nebulizer, ventilator, and hemodialysis device [6, 9].

*S. paucimobilis* is an opportunistic pathogen that rarely causes infection in humans because of its low virulence. It has a unique sphingoglycolipid in the cell wall and lacks the lipopolysaccharide component along with its endotoxin activity. This could be the explanation to the low virulence of this organism [4]. The majority of* S. paucimobilis* infection is associated with various comorbidities and immunodeficiencies, such as diabetes mellitus, malignancy, alcoholism, liver cirrhosis, end-stage renal disease, chronic obstructive pulmonary disease, burn injury, and acquired immunodeficiency syndrome, and is also associated with patients with indwelling catheters or devices [4]. There were also growing reports of infection in immunocompetent patient [11, 12].* S. paucimobilis* has been reported in a variety of communities and hospital infections such as bacteremia, catheter-related sepsis, meningitis, peritonitis, cutaneous infection, adenitis, septic arthritis, osteomyelitis, endophthalmitis, visceral abscesses, and diarrheal disease [10, 13–16]. The patient in this case report had all of the conditions associated with* S. paucimobilis* infection such sepsis due to diabetic foot ulcer, multiple comorbidities: diabetes mellitus, colonic tuberculosis, end-stage renal disease, and indwelling catheters for central venous catheter and hemodialysis. Severe infection and septic shock have been described particularly in immunocompromised patients [2, 4, 7], but only one case of death has been reported to be related to* S. paucimobilis* infection, which occurred in a premature neonate with septic shock [8]. This is the first reported lethal case of* S. paucimobilis* bacteremia in an adult patient.

*S. paucimobilis* is usually susceptible to carbapenems, aminoglycosides, trimethoprim sulfamethoxazole, and piperacillin/tazobactam and resistant to penicillins and first-generation cephalosporins. Its resistance to penicillins and first-generation cephalosporins is due to the production of chromosomally encoded beta-lactamase production [17]. The susceptibility to third-generation cephalosporins and fluoroquinolones is variable [1, 16]. A study reported that* S. paucimobilis* was resistant to amikacin, ceftazidime, and fluoroquinolones [9], while in another study it was resistant to cefoxitin and ceftazidime [18] and to cefotaxime and amikacin [19]. As there is no definitive guidelines for antimicrobial therapy for* S. paucimobilis* infections, treatment is done with individualized antibiotic therapy according to the in vitro susceptibility profile of clinical isolate [9, 18]. A study recommends the use of imipenem or aminoglycoside with a third-generation cephalosporin as the antibiotic regimen of choice in the treatment of* S. paucimobilis* [1].* S. paucimobilis* in this case was resistant to the antibiotics; it is usually susceptible to aminoglycosides, fluoroquinolones, trimethoprim sulfamethoxazole, and some third-generation cephalosporins. This susceptibility profile showed multidrug resistance, which poses new threat to the antibiotic therapy of this infection. This multidrug resistant* S. paucimobilis* would make use of aminoglycoside and third-generation cephalosporin as empiric therapy is inappropriate. Another fact in this case report was the result of blood culture that came out after the patient had passed away, so the antibiotics could not be changed according to the result. This delay would have resulted in the failure of antibiotic therapy and led to mortality of the patient.

## 4. Conclusion

We have reported the first lethal case of patient with* S. paucimobilis* bacteremia and multiple comorbidities: diabetes mellitus, colonic tuberculosis, end-stage renal disease, and indwelling catheters for central venous catheter and hemodialysis.* S. paucimobilis* was resistant to the antibiotics and it is usually susceptible to aminoglycosides, fluoroquinolones, trimethoprim sulfamethoxazole, and some third-generation cephalosporins.

## Figures and Tables

**Table 1:** The microorganism culture obtained from the patient.

	14th day	22th day			29th day	
Specimen	Blood	Blood	Sputum		Blood	
Isolate	Negative	Negative	*A. baumanii*		*S. paucimobilis*	
Susceptibility			Cefazoline	R	Cefazoline	R
			Cefepime	R	Cefepime	R
			Cefoperazone	R	Cefpirome	R
			**Cefoperazone sulbactam**	S	**Cefoperazone**	S
			Ceftazidime	R	**Cefoperazone sulbactam**	S
			Ceftriaxone	R	Ceftazidime	R
			Ampicillin	R	Ceftriaxone	R
			Piperacillin tazobactam	R	Ampicillin	R
			Ampicillin sulbactam	R	Ampicillin sulbactam	I
			**Fosfomycin**	S	Fosfomycin	R
			Imipenem	R	**Imipenem**	S
			Meropenem	R	Meropenem	I
			Tigecycline	R	**Tigecycline**	S
			Aztreonam	R	Aztreonam	R
			Levofloxacin	R	Levofloxacin	R
			Ciprofloxacin	R	Ciprofloxacin	R
			**Trimethoprim sulfamethoxazole**	S	Trimethoprim sulfamethoxazole	R
			Gentamicin	R	Gentamicin	R
			**Amikacin**	S	Amikacin	R
